# (*E*)-5-(2-Chloro­phen­yl)-7-ethyl-2-oxo-2,3-dihydro-1*H*-thieno[2,3-*e*][1,4]diazepin-4-ium 2,4,6-trinitro­phenolate

**DOI:** 10.1107/S1600536812002607

**Published:** 2012-01-25

**Authors:** Richard Betz, Thomas Gerber, Eric Hosten, Alaloor S. Dayananda, Hemmige S. Yathirajan, A. R. Ramesha

**Affiliations:** a Nelson Mandela Metropolitan University, Summerstrand Campus, Department of Chemistry, University Way, Summerstrand, PO Box 77000, Port Elizabeth 6031, South Africa; b University of Mysore, Department of Studies in Chemistry, Manasagangotri, Mysore 570 006, India; c R. L. Fine Chem., Bangalore 560 064, India

## Abstract

In the title molecular salt, C_15_H_14_ClN_2_OS^+^·C_6_H_2_N_3_O_7_^−^, protonation occurred on the double-bonded N atom. One of the nitro groups shows slight disorder over two orientations, with an occupancy ratio of 0.91:0.09. In the crystal, classical N—H⋯O hydrogen bonds, as well as C—H⋯O contacts connect the components into a three-dimensional network. The seven-membered ring adopts a boat-like conformation. The least-squares plane defined by its non-H atoms encloses an angle of 38.99 (6)° with the benzene ring bonded to it.

## Related literature

For pharmaceutical background to benzodiazepines, see: Robol *et al.* (1996 ▶); Evans *et al.* (2001 ▶). For related structures, see: Scammells *et al.* (2001 ▶); Jasinski *et al.* (2010 ▶). For graph-set analysis of hydrogen bonds, see: Etter *et al.* (1990 ▶); Bernstein *et al.* (1995 ▶). For puckering analysis, see: Cremer & Pople (1975 ▶).
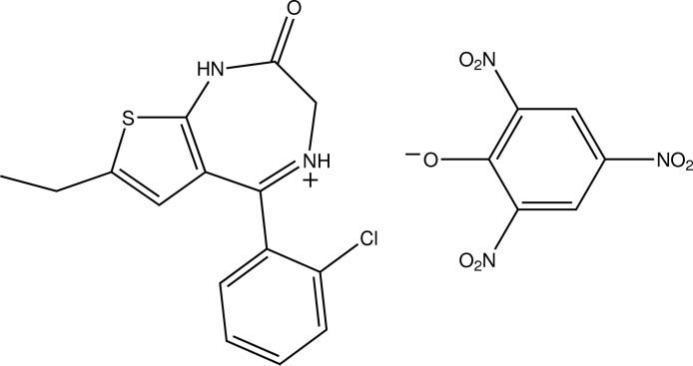


## Experimental

### 

#### Crystal data

C_15_H_14_ClN_2_OS^+^·C_6_H_2_N_3_O_7_^−^
*M* *_r_* = 533.90
Monoclinic, 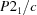

*a* = 10.5704 (2) Å
*b* = 20.0667 (5) Å
*c* = 11.3741 (2) Å
β = 110.666 (1)°
*V* = 2257.35 (8) Å^3^
*Z* = 4
Mo *K*α radiation
μ = 0.32 mm^−1^
*T* = 200 K
0.59 × 0.49 × 0.36 mm

#### Data collection

Bruker APEXII CCD diffractometer
Absorption correction: multi-scan (*SADABS*; Bruker, 2008 ▶). *T*_min_ = 0.807, *T*_max_ = 0.892
21254 measured reflections
5607 independent reflections
5073 reflections with *I* > 2σ(*I*)
*R*_int_ = 0.013

#### Refinement

*R*[*F* ^2^ > 2σ(*F* ^2^)] = 0.031
*wR*(*F* ^2^) = 0.081
*S* = 1.05
5607 reflections
343 parameters
H atoms treated by a mixture of independent and constrained refinement
Δρ_max_ = 0.38 e Å^−3^
Δρ_min_ = −0.24 e Å^−3^

### 

Data collection: *APEX2* (Bruker, 2010 ▶); cell refinement: *SAINT* (Bruker, 2010 ▶); data reduction: *SAINT*; program(s) used to solve structure: *SHELXS97* (Sheldrick, 2008 ▶); program(s) used to refine structure: *SHELXL97* (Sheldrick, 2008 ▶); molecular graphics: *ORTEP-3 for Windows* (Farrugia, 1997 ▶) and *Mercury* (Macrae *et al.*, 2008 ▶); software used to prepare material for publication: *SHELXL97* and *PLATON* (Spek, 2009 ▶).

## Supplementary Material

Crystal structure: contains datablock(s) I, global. DOI: 10.1107/S1600536812002607/pk2384sup1.cif

Supplementary material file. DOI: 10.1107/S1600536812002607/pk2384Isup2.cdx

Structure factors: contains datablock(s) I. DOI: 10.1107/S1600536812002607/pk2384Isup3.hkl

Supplementary material file. DOI: 10.1107/S1600536812002607/pk2384Isup4.cml

Additional supplementary materials:  crystallographic information; 3D view; checkCIF report

## Figures and Tables

**Table 1:** Hydrogen-bond geometry (Å, °)

*D*—H⋯*A*	*D*—H	H⋯*A*	*D*⋯*A*	*D*—H⋯*A*
N1—H71⋯O31	0.870 (19)	1.865 (19)	2.6847 (13)	156.3 (17)
N2—H72⋯O31^i^	0.836 (18)	2.047 (18)	2.8331 (13)	156.4 (16)
N2—H72⋯O362^i^	0.836 (18)	2.400 (17)	2.9495 (16)	123.9 (14)
C2—H2*A*⋯O321	0.99	2.49	3.2465 (16)	133
C2—H2*B*⋯O342^ii^	0.99	2.48	3.2505 (17)	134
C23—H23⋯O321^iii^	0.95	2.51	3.4193 (18)	161

Symmetry codes: (i) 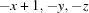; (ii) 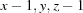; (iii) 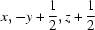.

**Fig. 1.**
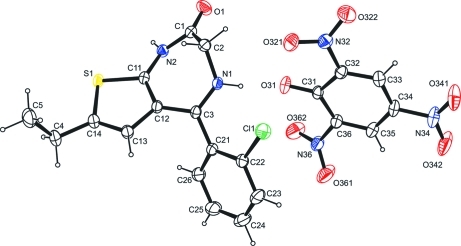
The molecular structure of the title compound, with anisotropic displacement ellipsoids (drawn at 50% probability level). For clarity, only the major component of the disordered nitro group is depicted.

**Fig. 2.**
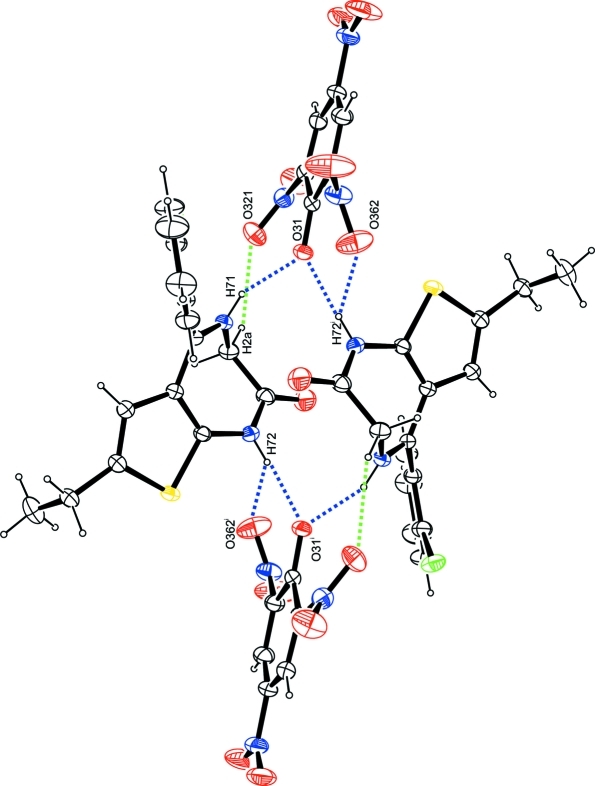
Intermolecular contacts, viewed approximately along [-1 -1 -1]. For clarity, only selected intermolecular contacts are depicted. Blue dashed lines show classical hydrogen bonds of the N–H···O type, green dashed lines C–H···O contacts. Symmetry operator: i -x + 1, -y, -z. Only the major component of the disordered nitro group is depicted.

## supplementary crystallographic information

### Comment

Five-atom heterocyclic fused benzodiazepine ring systems occupy a prominent place among drugs for treatment of central nervous system (*CNS*) disorders (Robol *et al.*, 1996; Evans *et al.*, 2001). The crystal structures of 6,7-dimethyl-5-phenyl-1*H*-thieno[2,3-*e*][1,4]diazepin-2(3*H*) -one (Scammells *et al.*, 2001) and 5,7-dimethyl-2,3-dihydro-1*H*-1,4- diazepin-4-ium picrate (Jasinski *et al.*, 2010) have been reported. In view of the importance of heterocyclic fused diazepine ring systems, the paper reports the crystal structure of the title compound.

The title compound is the picrate salt of (*E*)-5-(2-chlorophenyl)-7- ethyl-1*H*-thieno[2,3-*e*][1,4]diazepin-2(3*H*)-one. Due to involvement of the free electron pair on the – formally – *sp*^3^ hybridized nitrogen atom in amide-type resonance, protonation occurred on the double-bonded nitrogen atom. According to a puckering analysis (Cremer & Pople, 1975), the seven-membered heterocycle adopts a boat-like conformation (*Q*_2_: 0.7754 (12) Å, *Q*_3_: 0.2281 (12) Å, φ_2_: 262.69 (9)°, φ_3_: 207.4 (3)°) (Fig. 1).

In the crystal structure, classical hydrogen bonds of the N–H···O type as well as C–H···O contacts whose range falls by more than 0.2 Å below the sum of van-der-Waals radii of the corresponding atoms are apparent. While the classical hydrogen bonds are supported by both nitrogen-bound H atoms and have the phenolic as well as nitrogen-bound oxygen atoms as acceptor, the C–H···O contacts exclusively apply the latter type of oxygen atoms as acceptors. The C–H···O contacts stem from both hydrogen atoms of the intracyclic methylene group as well as the hydrogen atom in *ortho* position to the chlorine atom. In terms of graph-set analysis (Etter *et al.*, 1990; Bernstein *et al.*, 1995), the descriptor for the classical hydrogen bonds is *DDD* on the unitary level (due to bifurcation of one of the classical hydrogen bonds). The same descriptor is necessary to describe the C–H···O contacts on the identical level. Metrical information about the contacts is summarized in Table 1. In total, the entities of the title compound are connected to a three-dimensional network. The shortest intercentroid distance between two aromatic systems was measured at 4.8063 (7) Å and is apparent between the thiophene moieties of two neighbouring molecules. (Fig. 2).

### Experimental

(*E*)-5-(2-Chlorophenyl)-7-ethyl-1*H*-thieno[2,3-*e*] [1,4]diazepin-2(3*H*)-one was obtained as a gift sample from *R. L*. Fine Chem., Bengaluru, India. (*E*)-5-(2-Chlorophenyl)-7-ethyl-1*H*-thieno [2,3-*e*][1,4]diazepin-2(3*H*)-one (3.04 g, 0.01 mol) was dissolved in 10 ml of methanol and picric acid (2.29 g, 0.01 mol) was also dissolved in 10 ml of methanol. Both solutions were mixed and stirred in a beaker at 333 K for 30 minutes. The mixture was kept aside for a day at room temperature. The salt formed was filtered and dried in a vaccum desiccator over phosphorous pentoxide. The compound was recrystallized from a mixture (*v*:*v* = 1:1) of DMSO and ethanol by slow evaporation (m.p: 518 K).

### Refinement

Carbon-bound H atoms were placed in calculated positions (C—H 0.95 Å for aromatic carbon atoms, C—H 0.99 Å for methylene groups) and were included in the refinement in the riding model approximation, with *U*(H) set to 1.2*U*_eq_(C). The H atoms of the methyl group were allowed to rotate with a fixed angle around the C—C bond (C—H 0.98 Å) to best fit the experimental electron density (HFIX 137 in the *SHELX* program suite (Sheldrick, 2008), with *U*(H) set to 1.5*U*_eq_(C). Both nitrogen-bound H atoms were located on a difference Fourier map and refined freely.

### Crystal data

**Table:**

C_15_H_14_ClN_2_OS^+^·C_6_H_2_N_3_O_7_^−^	*F*(000) = 1096
*M**_r_* = 533.90	*D*_x_ = 1.571 Mg m^−^^3^
Monoclinic, *P*2_1_/*c*	Melting point: 518 K
Hall symbol: -P 2ybc	Mo *K*α radiation, λ = 0.71073 Å
*a* = 10.5704 (2) Å	Cell parameters from 9909 reflections
*b* = 20.0667 (5) Å	θ = 2.9–28.3°
*c* = 11.3741 (2) Å	µ = 0.32 mm^−^^1^
β = 110.666 (1)°	*T* = 200 K
*V* = 2257.35 (8) Å^3^	Block, brown
*Z* = 4	0.59 × 0.49 × 0.36 mm

### Data collection

**Table:**

Bruker APEXII CCD diffractometer	5607 independent reflections
Radiation source: fine-focus sealed tube	5073 reflections with *I* > 2σ(*I*)
graphite	*R*_int_ = 0.013
φ and ω scans	θ_max_ = 28.3°, θ_min_ = 2.0°
Absorption correction: multi-scan (*SADABS*; Bruker, 2008).	*h* = −13→14
*T*_min_ = 0.807, *T*_max_ = 0.892	*k* = −26→26
21254 measured reflections	*l* = −15→15

### Refinement

**Table:**

Refinement on *F*^2^	Primary atom site location: structure-invariant direct methods
Least-squares matrix: full	Secondary atom site location: difference Fourier map
*R*[*F*^2^ > 2σ(*F*^2^)] = 0.031	Hydrogen site location: inferred from neighbouring sites
*wR*(*F*^2^) = 0.081	H atoms treated by a mixture of independent and constrained refinement
*S* = 1.05	*w* = 1/[σ^2^(*F*_o_^2^) + (0.0349*P*)^2^ + 1.1718*P*] where *P* = (*F*_o_^2^ + 2*F*_c_^2^)/3
5607 reflections	(Δ/σ)_max_ < 0.001
343 parameters	Δρ_max_ = 0.38 e Å^−^^3^
0 restraints	Δρ_min_ = −0.24 e Å^−^^3^

### Fractional atomic coordinates and isotropic or equivalent isotropic displacement parameters (Å^2^)

**Table:**

	*x*	*y*	*z*	*U*_iso_*/*U*_eq_	Occ. (<1)
Cl1	0.57956 (3)	0.230591 (16)	0.27567 (3)	0.03062 (8)	
S1	0.08122 (3)	0.038903 (15)	−0.15866 (3)	0.02327 (8)	
O1	0.53537 (10)	0.04170 (5)	−0.16774 (10)	0.0361 (2)	
O31	0.75141 (9)	0.09665 (4)	0.21426 (8)	0.02763 (19)	
O321	0.75835 (10)	0.21026 (6)	0.09075 (9)	0.0377 (2)	
O322	0.96274 (13)	0.23796 (7)	0.11380 (11)	0.0495 (3)	
O341	1.27262 (12)	0.24856 (6)	0.54001 (12)	0.0505 (3)	
O342	1.25204 (13)	0.17919 (7)	0.67751 (12)	0.0607 (4)	
N36	0.84539 (12)	0.04459 (6)	0.46335 (11)	0.0328 (3)	
O361	0.8483 (2)	0.04566 (12)	0.57264 (12)	0.0591 (7)	0.913 (5)
O362	0.79227 (19)	−0.00036 (7)	0.39045 (12)	0.0432 (5)	0.913 (5)
O363	0.8925 (12)	0.0218 (6)	0.5586 (12)	0.023 (3)*	0.087 (5)
O364	0.7289 (16)	0.0258 (8)	0.3784 (12)	0.037 (4)*	0.087 (5)
N1	0.49406 (10)	0.12534 (5)	0.07439 (9)	0.01965 (19)	
H71	0.5767 (19)	0.1274 (9)	0.1273 (17)	0.040 (5)*	
N2	0.34466 (10)	0.03182 (5)	−0.12197 (9)	0.0216 (2)	
H72	0.3224 (17)	−0.0023 (9)	−0.1669 (16)	0.033 (4)*	
N32	0.87981 (11)	0.21070 (6)	0.15093 (10)	0.0277 (2)	
N34	1.21470 (12)	0.20290 (6)	0.57125 (12)	0.0375 (3)	
C1	0.45809 (12)	0.06515 (6)	−0.12171 (11)	0.0229 (2)	
C2	0.47774 (12)	0.13264 (6)	−0.05765 (11)	0.0228 (2)	
H2A	0.5588	0.1545	−0.0645	0.027*	
H2B	0.3985	0.1613	−0.1002	0.027*	
C3	0.39456 (11)	0.11469 (5)	0.11481 (10)	0.0186 (2)	
C4	−0.11953 (12)	0.08838 (7)	−0.07592 (13)	0.0272 (3)	
H4A	−0.1330	0.1015	0.0028	0.033*	
H4B	−0.1627	0.0444	−0.1017	0.033*	
C5	−0.18803 (14)	0.13922 (8)	−0.17752 (15)	0.0399 (3)	
H5A	−0.1502	0.1835	−0.1498	0.060*	
H5B	−0.2854	0.1397	−0.1933	0.060*	
H5C	−0.1727	0.1273	−0.2549	0.060*	
C11	0.24806 (11)	0.05778 (6)	−0.07911 (10)	0.0194 (2)	
C12	0.26416 (11)	0.09611 (6)	0.02726 (10)	0.0198 (2)	
C13	0.13619 (12)	0.10916 (6)	0.04104 (11)	0.0230 (2)	
H13	0.1277	0.1342	0.1088	0.028*	
C14	0.02920 (12)	0.08249 (6)	−0.05130 (11)	0.0232 (2)	
C21	0.42065 (11)	0.11893 (6)	0.25106 (11)	0.0210 (2)	
C22	0.50465 (12)	0.16687 (6)	0.33080 (11)	0.0243 (2)	
C23	0.52897 (15)	0.16634 (8)	0.45900 (13)	0.0368 (3)	
H23	0.5859	0.1992	0.5119	0.044*	
C24	0.47029 (17)	0.11802 (9)	0.50923 (13)	0.0428 (4)	
H24	0.4878	0.1174	0.5971	0.051*	
C25	0.38611 (16)	0.07037 (8)	0.43273 (14)	0.0377 (3)	
H25	0.3456	0.0373	0.4678	0.045*	
C26	0.36134 (13)	0.07120 (7)	0.30489 (12)	0.0283 (3)	
H26	0.3028	0.0387	0.2526	0.034*	
C31	0.85448 (11)	0.12197 (6)	0.29523 (11)	0.0208 (2)	
C32	0.92952 (12)	0.17741 (6)	0.27217 (11)	0.0225 (2)	
C33	1.04736 (12)	0.20209 (6)	0.35838 (12)	0.0256 (2)	
H33	1.0953	0.2371	0.3363	0.031*	
C34	1.09452 (12)	0.17498 (6)	0.47757 (12)	0.0267 (3)	
C35	1.02603 (13)	0.12448 (6)	0.51186 (12)	0.0280 (3)	
H35	1.0569	0.1080	0.5955	0.034*	
C36	0.91220 (12)	0.09847 (6)	0.42255 (11)	0.0244 (2)	

### Atomic displacement parameters (Å^2^)

**Table:**

	*U*^11^	*U*^22^	*U*^33^	*U*^12^	*U*^13^	*U*^23^
Cl1	0.03397 (16)	0.02676 (15)	0.03248 (16)	−0.01114 (12)	0.01341 (13)	−0.00916 (11)
S1	0.01661 (13)	0.02786 (15)	0.02118 (14)	−0.00278 (10)	0.00153 (10)	−0.00509 (10)
O1	0.0307 (5)	0.0438 (6)	0.0402 (5)	−0.0040 (4)	0.0204 (4)	−0.0127 (4)
O31	0.0212 (4)	0.0225 (4)	0.0289 (4)	−0.0008 (3)	−0.0038 (3)	−0.0028 (3)
O321	0.0293 (5)	0.0464 (6)	0.0305 (5)	0.0064 (4)	0.0020 (4)	0.0102 (4)
O322	0.0465 (6)	0.0593 (7)	0.0426 (6)	−0.0143 (6)	0.0155 (5)	0.0147 (5)
O341	0.0336 (6)	0.0361 (6)	0.0629 (8)	−0.0136 (5)	−0.0065 (5)	−0.0026 (5)
O342	0.0493 (7)	0.0693 (9)	0.0367 (6)	−0.0165 (6)	−0.0183 (5)	0.0039 (6)
N36	0.0317 (6)	0.0376 (6)	0.0242 (5)	−0.0089 (5)	0.0037 (4)	0.0028 (5)
O361	0.0699 (12)	0.0805 (14)	0.0235 (6)	−0.0390 (11)	0.0124 (6)	−0.0028 (7)
O362	0.0621 (11)	0.0307 (7)	0.0354 (6)	−0.0185 (7)	0.0154 (6)	−0.0032 (5)
N1	0.0162 (4)	0.0225 (5)	0.0180 (4)	−0.0010 (4)	0.0032 (4)	−0.0027 (3)
N2	0.0201 (5)	0.0224 (5)	0.0212 (5)	−0.0016 (4)	0.0061 (4)	−0.0056 (4)
N32	0.0303 (5)	0.0265 (5)	0.0250 (5)	0.0003 (4)	0.0082 (4)	0.0009 (4)
N34	0.0264 (6)	0.0322 (6)	0.0397 (7)	−0.0035 (5)	−0.0060 (5)	−0.0066 (5)
C1	0.0205 (5)	0.0290 (6)	0.0179 (5)	−0.0004 (4)	0.0052 (4)	−0.0012 (4)
C2	0.0228 (5)	0.0253 (5)	0.0201 (5)	−0.0040 (4)	0.0074 (4)	−0.0002 (4)
C3	0.0166 (5)	0.0174 (5)	0.0194 (5)	0.0000 (4)	0.0035 (4)	−0.0028 (4)
C4	0.0158 (5)	0.0295 (6)	0.0344 (6)	−0.0011 (4)	0.0065 (5)	−0.0028 (5)
C5	0.0244 (6)	0.0466 (8)	0.0456 (8)	0.0098 (6)	0.0083 (6)	0.0099 (7)
C11	0.0168 (5)	0.0203 (5)	0.0185 (5)	−0.0011 (4)	0.0030 (4)	−0.0006 (4)
C12	0.0163 (5)	0.0213 (5)	0.0199 (5)	−0.0009 (4)	0.0041 (4)	−0.0025 (4)
C13	0.0186 (5)	0.0248 (5)	0.0247 (5)	0.0001 (4)	0.0066 (4)	−0.0043 (4)
C14	0.0177 (5)	0.0242 (5)	0.0263 (6)	0.0001 (4)	0.0059 (4)	−0.0011 (4)
C21	0.0184 (5)	0.0240 (5)	0.0195 (5)	−0.0013 (4)	0.0052 (4)	−0.0040 (4)
C22	0.0218 (5)	0.0271 (6)	0.0237 (6)	−0.0050 (4)	0.0077 (4)	−0.0051 (4)
C23	0.0376 (7)	0.0466 (8)	0.0237 (6)	−0.0133 (6)	0.0077 (5)	−0.0128 (6)
C24	0.0486 (9)	0.0593 (10)	0.0206 (6)	−0.0123 (8)	0.0125 (6)	−0.0044 (6)
C25	0.0406 (8)	0.0459 (8)	0.0302 (7)	−0.0110 (6)	0.0168 (6)	0.0014 (6)
C26	0.0278 (6)	0.0305 (6)	0.0267 (6)	−0.0073 (5)	0.0099 (5)	−0.0036 (5)
C31	0.0171 (5)	0.0210 (5)	0.0218 (5)	0.0022 (4)	0.0037 (4)	−0.0029 (4)
C32	0.0212 (5)	0.0226 (5)	0.0215 (5)	0.0022 (4)	0.0049 (4)	−0.0002 (4)
C33	0.0204 (5)	0.0226 (5)	0.0317 (6)	−0.0009 (4)	0.0065 (5)	−0.0029 (5)
C34	0.0185 (5)	0.0257 (6)	0.0283 (6)	−0.0011 (4)	−0.0014 (5)	−0.0056 (5)
C35	0.0260 (6)	0.0291 (6)	0.0223 (5)	−0.0002 (5)	0.0005 (5)	−0.0009 (5)
C36	0.0227 (5)	0.0246 (6)	0.0231 (5)	−0.0023 (4)	0.0044 (5)	−0.0004 (4)

### Geometric parameters (Å, °)

**Table:**

Cl1—C22	1.7337 (13)	C4—C5	1.5199 (19)
S1—C11	1.7164 (11)	C4—H4A	0.9900
S1—C14	1.7415 (12)	C4—H4B	0.9900
O1—C1	1.2102 (15)	C5—H5A	0.9800
O31—C31	1.2590 (14)	C5—H5B	0.9800
O321—N32	1.2234 (15)	C5—H5C	0.9800
O322—N32	1.2277 (15)	C11—C12	1.3925 (15)
O341—N34	1.2222 (18)	C12—C13	1.4396 (15)
O342—N34	1.2273 (18)	C13—C14	1.3528 (16)
N36—O363	1.118 (12)	C13—H13	0.9500
N36—O362	1.2196 (16)	C21—C26	1.3985 (17)
N36—O361	1.2330 (19)	C21—C22	1.4029 (16)
N36—O364	1.323 (14)	C22—C23	1.3885 (18)
N36—C36	1.4542 (16)	C23—C24	1.379 (2)
N1—C3	1.3056 (15)	C23—H23	0.9500
N1—C2	1.4574 (15)	C24—C25	1.384 (2)
N1—H71	0.870 (19)	C24—H24	0.9500
N2—C1	1.3720 (15)	C25—C26	1.3836 (18)
N2—C11	1.3795 (15)	C25—H25	0.9500
N2—H72	0.836 (18)	C26—H26	0.9500
N32—C32	1.4534 (15)	C31—C36	1.4380 (16)
N34—C34	1.4519 (15)	C31—C32	1.4431 (16)
C1—C2	1.5170 (17)	C32—C33	1.3771 (16)
C2—H2A	0.9900	C33—C34	1.3803 (18)
C2—H2B	0.9900	C33—H33	0.9500
C3—C12	1.4358 (15)	C34—C35	1.3793 (18)
C3—C21	1.4776 (15)	C35—C36	1.3745 (17)
C4—C14	1.5011 (16)	C35—H35	0.9500
			
C11—S1—C14	92.26 (5)	C12—C11—S1	111.57 (8)
O363—N36—O362	107.3 (6)	C11—C12—C3	122.62 (10)
O362—N36—O361	122.68 (13)	C11—C12—C13	111.32 (10)
O363—N36—O364	124.9 (9)	C3—C12—C13	125.89 (10)
O361—N36—O364	115.2 (6)	C14—C13—C12	113.81 (10)
O363—N36—C36	120.9 (6)	C14—C13—H13	123.1
O362—N36—C36	119.46 (11)	C12—C13—H13	123.1
O361—N36—C36	117.83 (12)	C13—C14—C4	130.33 (11)
O364—N36—C36	114.2 (6)	C13—C14—S1	111.02 (9)
C3—N1—C2	124.21 (10)	C4—C14—S1	118.55 (9)
C3—N1—H71	120.1 (12)	C26—C21—C22	117.76 (11)
C2—N1—H71	115.7 (12)	C26—C21—C3	118.24 (10)
C1—N2—C11	124.78 (10)	C22—C21—C3	123.97 (11)
C1—N2—H72	117.2 (11)	C23—C22—C21	120.93 (12)
C11—N2—H72	116.2 (11)	C23—C22—Cl1	116.52 (10)
O321—N32—O322	123.13 (12)	C21—C22—Cl1	122.52 (9)
O321—N32—C32	118.95 (11)	C24—C23—C22	119.79 (13)
O322—N32—C32	117.92 (11)	C24—C23—H23	120.1
O341—N34—O342	123.85 (12)	C22—C23—H23	120.1
O341—N34—C34	118.31 (12)	C23—C24—C25	120.57 (13)
O342—N34—C34	117.84 (13)	C23—C24—H24	119.7
O1—C1—N2	122.16 (12)	C25—C24—H24	119.7
O1—C1—C2	123.78 (11)	C26—C25—C24	119.56 (13)
N2—C1—C2	114.05 (10)	C26—C25—H25	120.2
N1—C2—C1	110.59 (10)	C24—C25—H25	120.2
N1—C2—H2A	109.5	C25—C26—C21	121.37 (12)
C1—C2—H2A	109.5	C25—C26—H26	119.3
N1—C2—H2B	109.5	C21—C26—H26	119.3
C1—C2—H2B	109.5	O31—C31—C36	123.67 (11)
H2A—C2—H2B	108.1	O31—C31—C32	124.57 (11)
N1—C3—C12	119.59 (10)	C36—C31—C32	111.76 (10)
N1—C3—C21	118.98 (10)	C33—C32—C31	124.39 (11)
C12—C3—C21	121.36 (10)	C33—C32—N32	116.33 (11)
C14—C4—C5	112.41 (11)	C31—C32—N32	119.27 (10)
C14—C4—H4A	109.1	C32—C33—C34	118.69 (12)
C5—C4—H4A	109.1	C32—C33—H33	120.7
C14—C4—H4B	109.1	C34—C33—H33	120.7
C5—C4—H4B	109.1	C35—C34—C33	121.57 (11)
H4A—C4—H4B	107.9	C35—C34—N34	119.12 (12)
C4—C5—H5A	109.5	C33—C34—N34	119.21 (12)
C4—C5—H5B	109.5	C36—C35—C34	118.68 (12)
H5A—C5—H5B	109.5	C36—C35—H35	120.7
C4—C5—H5C	109.5	C34—C35—H35	120.7
H5A—C5—H5C	109.5	C35—C36—C31	124.71 (11)
H5B—C5—H5C	109.5	C35—C36—N36	116.33 (11)
N2—C11—C12	129.56 (10)	C31—C36—N36	118.94 (10)
N2—C11—S1	118.77 (8)		
			
C11—N2—C1—O1	−175.18 (12)	C22—C23—C24—C25	−0.7 (3)
C11—N2—C1—C2	5.61 (16)	C23—C24—C25—C26	0.3 (3)
C3—N1—C2—C1	−77.66 (14)	C24—C25—C26—C21	0.6 (2)
O1—C1—C2—N1	−117.45 (13)	C22—C21—C26—C25	−1.1 (2)
N2—C1—C2—N1	61.75 (13)	C3—C21—C26—C25	177.14 (13)
C2—N1—C3—C12	12.62 (17)	O31—C31—C32—C33	−175.40 (12)
C2—N1—C3—C21	−170.29 (10)	C36—C31—C32—C33	4.22 (17)
C1—N2—C11—C12	−41.65 (19)	O31—C31—C32—N32	4.86 (18)
C1—N2—C11—S1	142.17 (10)	C36—C31—C32—N32	−175.52 (10)
C14—S1—C11—N2	177.15 (10)	O321—N32—C32—C33	−152.12 (12)
C14—S1—C11—C12	0.32 (9)	O322—N32—C32—C33	28.00 (17)
N2—C11—C12—C3	−0.8 (2)	O321—N32—C32—C31	27.64 (17)
S1—C11—C12—C3	175.59 (9)	O322—N32—C32—C31	−152.24 (12)
N2—C11—C12—C13	−176.29 (12)	C31—C32—C33—C34	−3.70 (19)
S1—C11—C12—C13	0.10 (13)	N32—C32—C33—C34	176.05 (11)
N1—C3—C12—C11	32.88 (17)	C32—C33—C34—C35	−0.45 (19)
C21—C3—C12—C11	−144.13 (12)	C32—C33—C34—N34	−176.78 (12)
N1—C3—C12—C13	−152.30 (12)	O341—N34—C34—C35	−178.51 (13)
C21—C3—C12—C13	30.68 (18)	O342—N34—C34—C35	1.4 (2)
C11—C12—C13—C14	−0.65 (15)	O341—N34—C34—C33	−2.1 (2)
C3—C12—C13—C14	−175.96 (11)	O342—N34—C34—C33	177.82 (14)
C12—C13—C14—C4	−175.42 (12)	C33—C34—C35—C36	3.5 (2)
C12—C13—C14—S1	0.88 (14)	N34—C34—C35—C36	179.86 (12)
C5—C4—C14—C13	99.93 (17)	C34—C35—C36—C31	−2.8 (2)
C5—C4—C14—S1	−76.14 (14)	C34—C35—C36—N36	178.98 (12)
C11—S1—C14—C13	−0.69 (10)	O31—C31—C36—C35	178.71 (12)
C11—S1—C14—C4	176.10 (10)	C32—C31—C36—C35	−0.92 (17)
N1—C3—C21—C26	−138.62 (12)	O31—C31—C36—N36	−3.08 (18)
C12—C3—C21—C26	38.41 (16)	C32—C31—C36—N36	177.29 (11)
N1—C3—C21—C22	39.47 (17)	O363—N36—C36—C35	−7.5 (9)
C12—C3—C21—C22	−143.50 (12)	O362—N36—C36—C35	−144.83 (16)
C26—C21—C22—C23	0.68 (19)	O361—N36—C36—C35	33.3 (2)
C3—C21—C22—C23	−177.42 (12)	O364—N36—C36—C35	173.3 (8)
C26—C21—C22—Cl1	−177.61 (10)	O363—N36—C36—C31	174.1 (8)
C3—C21—C22—Cl1	4.29 (17)	O362—N36—C36—C31	36.8 (2)
C21—C22—C23—C24	0.2 (2)	O361—N36—C36—C31	−145.06 (19)
Cl1—C22—C23—C24	178.56 (13)	O364—N36—C36—C31	−5.1 (9)

### Hydrogen-bond geometry (Å, °)

**Table:**

*D*—H···*A*	*D*—H	H···*A*	*D*···*A*	*D*—H···*A*
N1—H71···O31	0.870 (19)	1.865 (19)	2.6847 (13)	156.3 (17)
N2—H72···O31^i^	0.836 (18)	2.047 (18)	2.8331 (13)	156.4 (16)
N2—H72···O362^i^	0.836 (18)	2.400 (17)	2.9495 (16)	123.9 (14)
C2—H2A···O321	0.99	2.49	3.2465 (16)	133
C2—H2B···O342^ii^	0.99	2.48	3.2505 (17)	134
C23—H23···O321^iii^	0.95	2.51	3.4193 (18)	161

Symmetry codes: (i) −*x*+1, −*y*, −*z*; (ii) *x*−1, *y*, *z*−1; (iii) *x*, −*y*+1/2, *z*+1/2.
